# Contribution of Intestinal Barrier Damage, Microbial Translocation and HIV-1 Infection Status to an Inflammaging Signature

**DOI:** 10.1371/journal.pone.0097171

**Published:** 2014-05-12

**Authors:** Amanda K. Steele, Eric J. Lee, Brian Vestal, Daniel Hecht, Zachary Dong, Eric Rapaport, John Koeppe, Thomas B. Campbell, Cara C. Wilson

**Affiliations:** 1 Department of Medicine, University of Colorado School of Medicine, Aurora, Colorado, United States of America; 2 Department of Immunology, University of Colorado School of Medicine, Aurora, Colorado, United States of America; 3 Colorado Biostatistics Consortium Research Consulting Laboratory, University of Colorado, Aurora, Colorado, United States of America; INSERM, France

## Abstract

**Background:**

Systemic inflammation is a characteristic of both HIV-1 infection and aging (“inflammaging”). Intestinal epithelial barrier damage (IEBD) and microbial translocation (MT) contribute to HIV-associated inflammation, but their impact on inflammaging remains unclear.

**Methods:**

Plasma biomarkers for IEBD (iFABP), MT (LPS, sCD14), T-cell activation (sCD27), and inflammation (hsCRP, IL-6) were measured in 88 HIV-1 uninfected (HIV^neg^) and 83 treated, HIV-1-infected (HIV^pos^) adults from 20–100 years old.

**Results:**

Age positively correlated with iFABP (r = 0.284, *p* = 0.008), sCD14 (r = 0.646, *p* = <0.0001) and LPS (r = 0.421, *p* = 0.0002) levels in HIV^neg^ but not HIV^pos^ subjects. Age also correlated with sCD27, hsCRP, and IL-6 levels regardless of HIV status. Middle-aged HIV^pos^ subjects had elevated plasma biomarker levels similar to or greater than those of elderly HIV^neg^ subjects with the exception of sCD14. Clustering analysis described an inflammaging phenotype (IP) based on iFABP, sCD14, sCD27, and hsCRP levels in HIV^neg^ subjects over 60 years of age. The IP in HIV^neg^ subjects was used to develop a classification model that was applied to HIV^pos^ subjects to determine whether HIV^pos^ subjects under 60 years of age were IP+. HIV^pos^ IP+ subjects were similar in age to IP- subjects but had a greater risk of cardiovascular disease (CVD) based on Framingham risk score (*p* =  0.01).

**Conclusions:**

We describe a novel IP that incorporates biomarkers of IEBD, MT, immune activation as well as inflammation. Application of this novel IP in HIV-infected subjects identified a group at higher risk of CVD.

## Introduction

Inflammation increases in an age-associated manner termed inflammaging, which is characterized by increased plasma levels of pro-inflammatory cytokines (IL-6, IL-15, IL-8), coagulation factors (D-dimer), and acute phase reactants (C-reactive protein (hsCRP) [1], [2]. In otherwise healthy elderly people, increased plasma levels of hsCRP and IL-6 have been linked to cardiovascular disease (CVD) [3], impaired mobility [4], cancer fatality [5], cognitive decline [2], and all-cause mortality [6]–[8]. Aging is also characterized by immunosenescence related changes that include: reduced CD4:CD8 T cell ratios; expansion of memory T cells; and increased expression of activation (CD38 and HLA-DR) and senescence (CD28-; CD57+) markers on CD4+ and CD8+ T cells [Reviewed in [9]]. Although inflammaging and immunosenescence are likely connected, the underlying mechanisms are multifaceted and incompletely defined.

The characteristics of age-associated inflammation are similar to inflammatory changes that occur during HIV-1 infection, including increased plasma IL-6 levels [10]. Despite receiving combination antiretroviral therapy (cART), plasma hsCRP and IL-6 remain significantly elevated in HIV-infected patients [11]. Similar to inflammaging, elevated IL-6 is associated with frailty [12] and increased risk of all-cause mortality [13] during treated HIV-infection. Given the increased lifespan of HIV-infected patients in the cART era, understanding whether inflammaging and HIV-associated inflammation are synergistic is critical [14]. However, it is unclear whether HIV-1 infection is an added risk factor for age-associated inflammation or whether HIV-associated inflammation is accelerating the inflammaging process [14].

HIV-associated inflammation has been linked to multiple factors including but not limited to: antigenic stimulation from the virus itself, viral proteins (e.g. gp120) that directly stimulate proinflammatory cytokine production, and immunodeficiency leading to reactivation of chronic pathogens (e.g. CMV). HIV-associated inflammation has also been linked to increased microbial translocation (MT), the movement of bacteria and their products across the intestinal epithelial barrier into the periphery [15]. HIV-associated MT is a result of intestinal epithelial barrier damage (IEBD) and intestinal CD4+ T-cell depletion [15]–[17]. A causal link between MT and systemic inflammation was established by Ferguson et al., who showed that LPS administration increased plasma concentrations of hsCRP and IL-6 in healthy volunteers [18]. Our first goal was to determine whether plasma biomarkers of IEBD and MT were associated with normal inflammaging and if so, to describe an inflammaging phenotype incorporating inflammatory and IEBD/MT markers. The second goal was to establish whether there was a synergistic interaction between HIV-1 infection status and age, and finally, to determine the factors associated with an inflammaged phenotype in HIV^pos^ subjects.

## Results

### Characterizing inflammaging in HIV^neg^ subjects

The demographics of 83 HIV^pos^ and 88 HIV^neg^ subjects enrolled in the study are summarized in Table 1. Both cohorts were predominately male (HIV^neg^ 63% vs. HIV^pos^ 83%) but there were significantly more women in the HIV^neg^ cohort (Table 1). There were also significantly more non-Caucasian subjects in the HIV^pos^ cohort (28%) than the HIV^neg^ cohort (15%). The groups differed in their median age: HIV^pos^ - 56 years (range: 24–81) vs. HIV^neg^ - 39 years (20–100) (*p* < 0.05). The age distribution and frequency of each cohort is shown in (Fig. S1). One notable difference in the age distribution between cohorts was the larger population of 20–30 year old donors in the HIV^neg^ cohort than was present in the HIV^pos^ cohort (Fig. S1). In addition, although the majority of subjects in both groups were within the same age range, the HIV^neg^ cohort included four subjects that were older than any of the HIV^pos^ subjects (Fig. S1).

**Table 1:** Clinical Characteristics.

	HIV^neg^	HIV^pos^	p value
***Number of subjects***	88	83	
men (n(%))	55 (63%)	69 (83%)	** 1
women (n(%))	33 (37%)	14 (17%)	
***Race***			
*Caucasian*	75 (85%)	60 (72%)	* 1
*Non-Caucasian (African America, Hispanic, Asian)*	13 (15%)	23 (28%)	
***median age (range)***	38.5 (20–100)	56 (24–81)	*** 2
***18*** *–* ***39yo (median (range)***	27 (20–39)	32 (24–39)	** 2
men (n(%))	26 (55%)	13 (86%)	* 1
women (n(%))	21 (45%)	2 (13%)	
***40*** *–* ***59yo (median (range)***	50 (40–57)	49 (40–58)	
men (n(%))	10 (67%)	26 (89%)	
women (n(%))	5 (33%)	3 (11%)	
***60+yo (median (range))***	69.5 (60–100)	64 (60–81)	** 2
men (n(%))	19 (73%)	30 (77%)	
women (n(%))	7 (27%)	9 (23%)	
			
***Median BMI (range)***	n.d.	25.1 (18.1–46.0)	
			
***Smokers (n(%))***	n.d.	34 (41%)	
***HIV Status***			
Viral Load (copies/mL)	–	< 48	
Viral load pre ART median (range) (N = 36; 43%)	–	94,200 (2258–1,466,650)	
CD4+ T cell (cells/mL)	–	595 (130–1400)	
Infection Duration (months) (N = 76; 92%)	–	107 (14–303)	
Time since first exposure to cART (months) (N = 65; 78%)	–	77 (10–280)	
INR CD4 <350 post cART (N = 56; 67%)^3^	–	6 (11%)	
median CD4 nadir (N = 60; 72%)	–	227 (11–798)	
CD4 nadir <200 (n(%)) (N = 60; 72%)	–	26 (43%)	
HCV positive (N = 70; 76%) (n (%))	n.d.	11 (16%)	

* < 0.05.

** 0.01–0.001.

*** < 0.0001.

1 Fisher's exact test

2 Mann-Whitney T test

3 Immunological non-responder (INR) status in patients where cART was documented for a minimum of 24 months. Median treatment time for these patients: 85.5 months (27–280).

**Figure 1:**
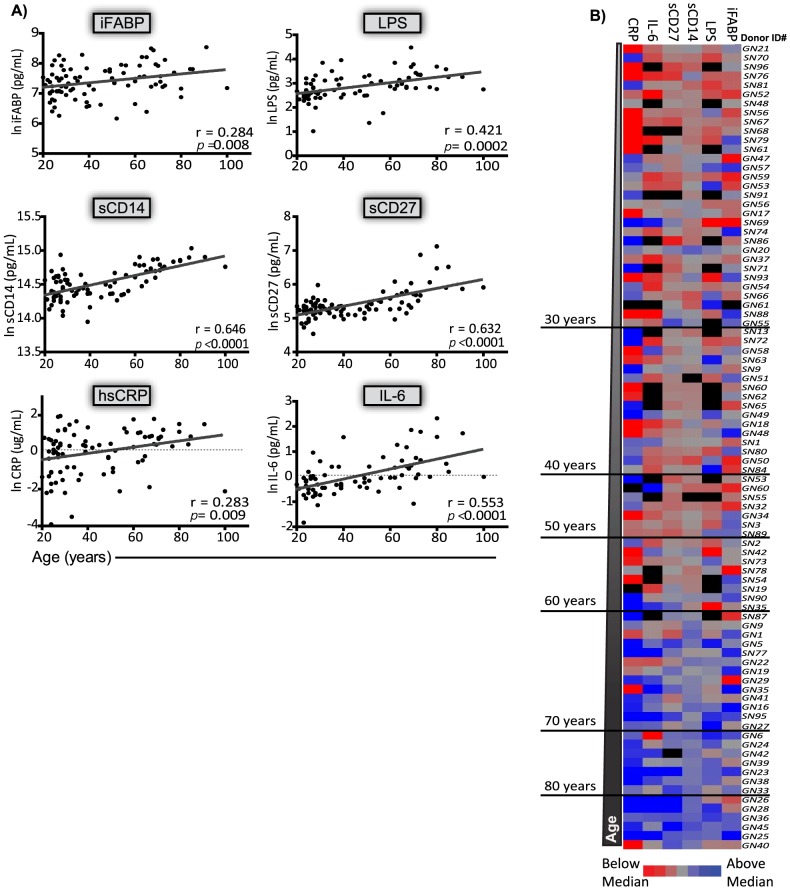
Age associations with plasma markers of MT and inflammation in HIVneg subjects. Plasma biomarkers were measured by ELISA in the plasma of subjects ranging from 20–100 years of age. (A) Individual associations between age and iFABP, LPS, sCD14, sCD27, hsCRP, and IL-6. Pearson correlations were conducted on natural log transformed plasma concentrations. The solid line indicates the best fit regression line for parameters that were significantly correlated with age. P < 0.05 was considered a significant association. The Pearson r and exact p-value for each association is shown. (B) Heat map showing the plasma concentrations of all six markers in individual subjects ordered from youngest to oldest. The untransformed plasma concentrations were median centered and plotted. Grey  =  the median value, Red  =  below median, Blue  =  above median, black  =  missing value. The heat map was normalized to the range for each individual marker so that the fold change represented by each color is consistent across markers.

To quantify IEBD, MT, and systemic inflammation we evaluated the plasma concentrations of intestinal fatty acid binding protein (iFABP; IEBD); LPS (direct MT) and sCD14 (indirect MT and monocyte activation); sCD27 (T cell activation); and hsCRP and IL-6 (systemic inflammation). In HIV^neg^ subjects, age correlated with levels of iFABP (r = 0.284, *p = 0.008*); LPS (r = 0.421, *p = 0.002*); sCD14 (r = 0.646, *p<0.0001*); sCD27(r = 0.632, *p<0.0001*); hsCRP (r = 0.283, *p = 0.009*); and IL-6 (r = 0.553; *p<0.001*) (Fig. 1A). The plasma concentrations of all the biomarkers were 1.4–2.5 times higher in the elderly subjects than in the young adults (Fig. S2). A heat map was used to visualize the pattern of biomarker expression for each subject. A gradient was observed within each biomarker from low to high levels as subject age increased (Fig. 1B; *vertical columns*). However, within an individual subject, few HIV^neg^ subjects had uniformly high or low biomarker levels (Fig. 1B; *horizontal rows*).

Using multivariable regression modeling (model 1), no association was detected between sex and the levels of iFABP, sCD14, sCD27, hsCRP, and IL-6 (Table S1). Men had slightly higher levels of LPS (coefficient: 0.35, *p*  = 0.01) that equated to 1.4 pg/mL. Given the high co-efficient of variation in the LPS assay, this difference is unlikely to be biologically meaningful (Table S1).

### Markers of immune activation and inflammation, but not IEBD or MT, were positively associated with age in HIV^pos^ subjects

**Figure 2:**
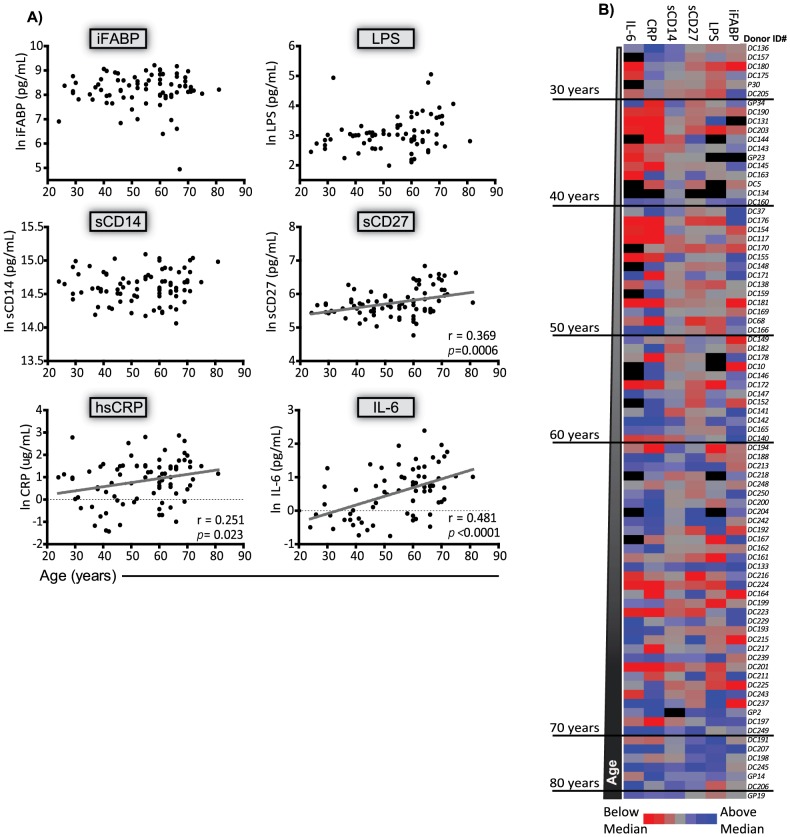
Age associations with plasma markers of MT and inflammation in HIVpos subjects. Plasma biomarkers were measured by ELISA in the plasma of subjects ranging from 24–81 years of age. (A) Individual associations between age iFABP, LPS, sCD14, sCD27, hsCRP, and IL-6. Pearson correlations were conducted on natural log transformed plasma concentrations. The solid line indicates the best fit regression line for parameters that were significantly correlated with age. P < 0.05 was considered a significant association. The Pearson r and exact p-value for each association is shown. (B) Heat map showing the plasma concentrations of all six markers in individual subjects ordered from youngest to oldest. The untransformed plasma concentrations were median centered and plotted. Grey  =  the median value, Red  =  below median, Blue  =  above median, black  =  missing value. The heat map was normalized to the range for each individual marker so that the fold change represented by each color is consistent across markers.

In HIV^pos^ subjects, iFABP, LPS, and sCD14 did not correlate with age (Fig. 2A). However, sCD27 (r = 0.369, *p = 0.0006*), hsCRP (r = 0.251; *p = 0.023*), and IL-6 (r = 0.481, *p*<0.0001) were significantly correlated with age (Fig. 2A). The age associations observed for sCD27, hsCRP, and IL6 remained significant after accounting for CD4+ T cell levels (Table S2). Since there was a greater proportion of 20–30 year old subjects in the HIV^neg^ cohort than the HIV^pos^ cohort, a series of diagnostic analyses were performed on the residuals from regression model 2 for each biomarker. These analyses did not identify any apparent outliers in the dataset. However, the subjects that could be considered most conspicuous (meaning they had the greatest impact on the regression results) were generally HIV^pos^, on the higher end of the age spectrum, and typically had high plasma levels of each biomarker that fell above the fitted regression line (data not shown). Therefore, we concluded that it was unlikely the disparity in the proportion of 20–30 year old subjects explained the lack of an age association for iFABP and LPS in the HIV^pos^ cohort.

In contrast to the HIV^neg^ subjects, the pattern of biomarker expression in the HIV^pos^ subjects did not show a clear gradient as the subjects' age increased (Fig. 2B; *vertical columns* compare to Fig. 1B). The most noticeable difference was that HIV^pos^ subjects had high levels of one or more of the plasma biomarkers at younger ages (Fig. 2B). Consistent with previous reports [17], [19]–[22], the geometric means of iFABP (2.6x; 2.8x), sCD14 (1.2x; 1.3x), sCD27 (1.4x; 1.4x), and hsCRP (1.7x; 3.1x) were significantly elevated in the young adult and middle aged HIV^pos^ subjects, respectively despite effective treatment with antiretroviral drugs (Fig. S3). This suggests that elevated levels of IEBD and MT markers associated with HIV infection could be masking more subtle age-associated changes. However, similar to the HIV^neg^ group, HIV^pos^ subjects rarely expressed uniformly high or low plasma concentrations of all six biomarkers within a given subject (Fig, 2B; *horizontal rows*).

### Associations between Biomarkers in HIV^neg^ and HIV^pos^ subjects

The associations between IEBD and MT markers and established inflammaging biomarkers, IL-6 and hsCRP, and with sCD27 were evaluated in both subject groups. In HIV^neg^ subjects, iFABP and LPS levels did not correlate with hsCRP or IL-6 but did correlate with sCD14 and sCD27 levels (Table 2). Although a direct correlation was not observed between markers of IEBD/MT and systemic inflammation, they could be indirectly linked via associations with cellular immune activation. Consistent with this hypothesis, a positive correlation was observed between sCD14 and sCD27 (r =  0.513, *p <0.001*) (Table 2). Furthermore, positive associations were observed between IL-6 and sCD14 (r =  0.288, *p* =  0.0155) and sCD27 (r =  0.6, *p* =  <0.001); and between sCD27 and hsCRP (r =  0.325, *p* =  0.0029) (Table 2).

**Table 2:** Plasma Biomarker Correlation Matrix in HIV^neg^ and HIV^pos^ Subjects.

*HIV^neg^ subjects*						
	***iFABP (pg/mL)***	***LPS (pg/mL)***	***sCD14 (pg/mL)***	***sCD27 (pg/mL)***	***hsCRP (ug/mL)***	***IL-6 (ng/mL)***
***iFABP (pg/mL)***	–	0.147 (0.2218)	0.347 * (0.0011)	0.230* (0.0351)	−0.128 (0.2422)	0.108 (0.3684)
***LPS (pg/mL)***		–	0.294* (0.0128)	0.248* (0.0386)	0.182 (0.1319)	0.166 (0.1735)
***sCD14 (pg/mL)***			–	0.513* (<0.0001)	0.149 (0.1797)	0.288* (0.0155)
***sCD27 (pg/mL)***				–	0.325* (0.0029)	0.600* (<0.0001)
***hsCRP (ug/mL)***					–	0.435* (0.0002)
***IL-6 (ng/mL)***						–

*HIV^pos^ subjects*						
	***iFABP (pg/mL)***	***LPS (pg/mL)***	***sCD14 (pg/mL)***	***sCD27 (pg/mL)***	***hsCRP (ug/mL)***	***IL-6 (ng/mL)***
***iFABP (pg/mL)***	–	−0.195 (0.096)	−0.019 (0.870)	−0.013 (0.912)	−0.041 (0.717)	−0.115 (0.355)
***LPS (pg/mL)***		–	0.186 (0.112)	0.225 (0.052)	0.206 (0.076)	0.209 (0.089)
***sCD14 (pg/mL)***			–	0.222* (0.046)	0.292* (0.008)	0.418* (<0.0001)
***sCD27 (pg/mL)***				–	0.198 (0.074)	0.505* (<0.0001)
***hsCRP (ug/mL)***					–	0.572* (<0.0001)
***IL-6 (ng/mL)***						–

Data is presented as r(p value).

*Indicates a significant correlation.

In the HIV^pos^ subjects, iFABP and LPS did not correlate with any other biomarkers (Table 2). LPS tended to have weak positive associations with sCD27 (r =  0.225; *p  =  0.052*); hsCRP (r =  0.206; *p* =  0.076); and IL-6 (r =  0.209; *p*  =  0.089) but none reached statistical significance in this cohort. However, significant associations were observed in HIV^pos^ subjects between sCD14 and hsCRP (r =  0.292; *p* =  0.008) and IL-6 (r =  0.418, *p* < 0.0001); and between sCD27 and IL-6 (r =  0.505; *p*< 0.0001) (Table 2).

### HIV^pos^ subjects have an inflammaged phenotype at a younger age despite cart

**Figure 3:**
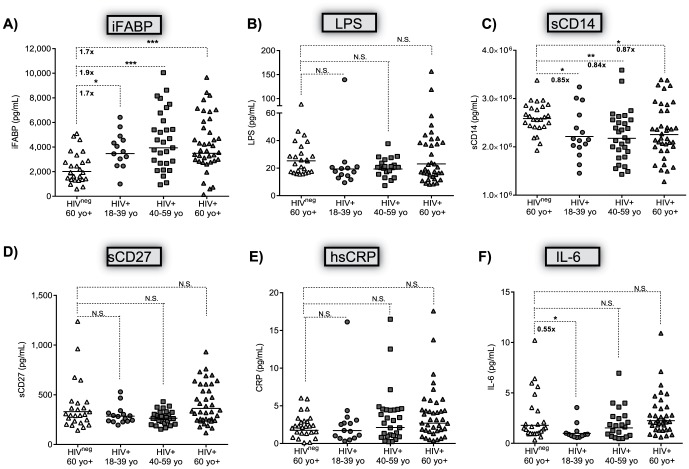
Comparing the plasma concentration of IEBD, MT, and inflammatory markers in elderly HIVneg subjects to HIVpos subjects stratified by age. Subjects were stratified into three age groups: young adult- 18–39 years of age; middle aged- 40–59 years of age; and elderly- >60 years of age. The biomarker concentrations were natural log transformed and compared between groups using an ANOVA, a p-value < 0.05 was considered significant. The Benjamini-Hochberg method was used to control false discovery rate (FDR) at the 0.05 level. The horizontal line indicates the geometric mean of each group. The fold-change is indicated when differences between the groups were significant. N.S. – not significant. * - *p* =  0.05–0.01; **- *p* =  0.0065; ***- *p* =  0.0002. Each group is represented by the same symbol throughout the figure: HIVneg subjects > 60 years of age- open triangle; HIVpos young adults- closed circle, HIVpos middle aged adults- closed square, HIVpos > 60 years- closed triangle. (A) iFABP, (B) LPS, (C) sCD14, (D) sCD27, (E) hsCRP, and (F) IL-6.

The biomarker levels in HIV^pos^ subjects of different age groups were compared to elderly HIV^neg^ subjects to determine whether the levels of inflammatory markers in HIV^pos^ subjects under 60 years of age were comparable to levels associated with HIV^neg^ inflammaging. HIV^pos^ subjects of all age groups had significantly higher iFABP levels than elderly HIV^neg^ donors (Fig. 3A; overall *p* value =  0.0002). The levels of LPS, sCD27, hsCRP in all age groups, and IL-6 by middle-age, in HIV^pos^ subjects were similar to elderly HIV^neg^ subjects (Figure 3B, D–F). However, elderly HIV^neg^ subjects had significantly higher plasma sCD14 levels (2.59×10^6^ pg/mL) than HIV^pos^ subjects of all ages (young: 2.21×10^6^ pg/mL; middle-aged: 2.18×10^6^ pg/mL; and elderly: 2.25×10^6^ pg/mL respectively) (Fig. 3C; overall *p* value =  0.0065). Excluding the four oldest HIV^neg^ subjects from the comparative analyses did not alter the conclusions presented (data not shown).

**Figure 4:**
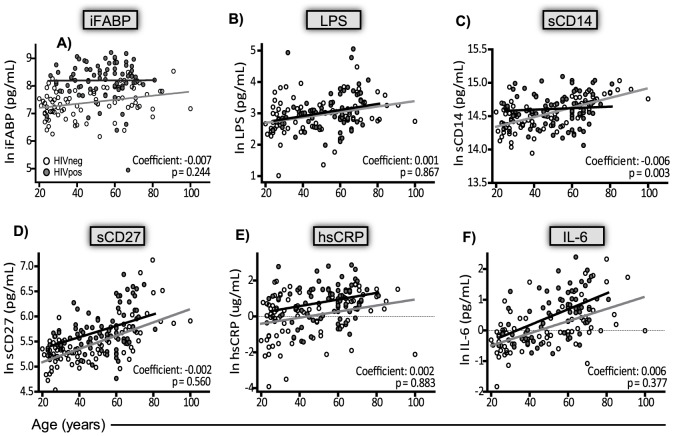
Multivariable regression models describing the HIV/age interaction for each plasma biomarker. Regression modeling was conducted on the natural log transformed plasma biomarker concentrations using HIV-status and age as covariates, and allowing an HIV/age interaction in the pooled HIVneg and HIVpos subjects. The best-fit line for the HIVpos (dark line, closed circles) and HIVneg (grey line, open circles) groups are shown for each biomarker. The HIV/age interaction coefficient and exact p-value are shown. A dotted horizontal line indicates zero on the natural log scale. (A) iFABP, (B) LPS, (C) sCD14, (D) sCD27, (E) hsCRP, and (F) IL6.

Regression analysis (model 2) was used to determine if HIV-1 infection status impacted the relationship between each biomarker and age. To quantify this, the HIV/age interaction was determined. In this context, the HIV/age interaction term is interpreted as the average increase or decrease in the expected yearly rate of change for each biomarker (slope of the line) attributable to HIV-status. The HIV/age interaction term was not significant for iFABP, LPS, sCD27, hsCRP, or IL-6 (Fig. 4 A–B, D–F). In contrast, sCD14 increased more slowly with age in the HIV^pos^ than HIV^neg^ subjects (coefficient: −0.006; *p  = 0.003*) (Fig. 4C). Additional regression modeling (model 3) indicated that HIV^pos^ subjects had biomarker levels equivalent to HIV^neg^ subjects that were older by 6–141 years (Table S1). The calculated difference between the biological and chronological age of HIV^pos^ subjects (141 years) based on iFABP levels was not biologically meaningful but does reinforce the importance of intestinal damage during HIV infection.

### Aggregate plasma biomarker expression identifies high and low inflammation phenotypes in HIV-infected donors irrespective of age

**Figure 5:**
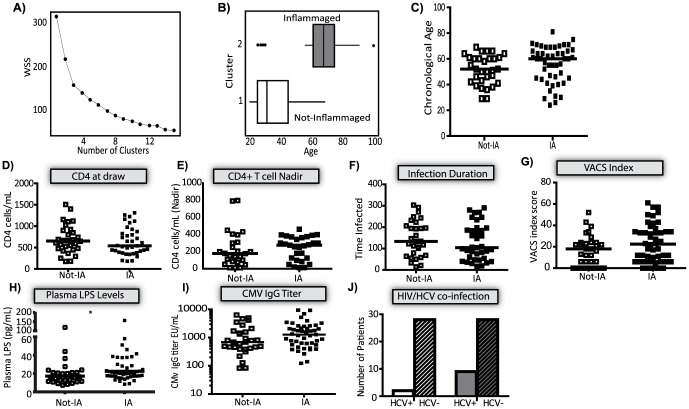
Identifying a plasma signature of inflammaging. The plasma concentrations of iFABP, sCD14, sCD27, hsCRP were incorporated into a k-means clustering analysis and linear discriminate function fit to the HIVneg donors. (A) WSS plot supporting a two cluster solution. (B) Age distribution of HIVneg subjects classified as IP+ (grey bar) or IP- (open bar). (C) The age distribution of IP+ (closed squares) or IP- (open squares) HIVpos subjects. For clinical parameters, the median (dot plots) or the event frequency (bar graph) in the HIVpos subjects is shown. In all cases the IP+ subjects are shown in closed squares or grey bars and compared to IP- subjects shown in open squares or open bars. Statistical significance was determined using the Mann-Whitney U test or Fisher's exact test as appropriate, p-value < 0.05 was significant. (D) CD4 T cell count at draw, (E) CD4+ T cell nadir, (F) Infection duration. (G) VACS index. (H) Plasma LPS concentration. To remain consistent with previous biomarker analysis: the plasma concentration was natural log transformed and a T-test was used to determine statistical significance. The horizontal line indicates the geometric mean. *-*p*  =  0.04 (I) CMV IgG titer in the plasma. (J) Frequency of HIV/HCV co-infection. Subjects that had a record of detectable hepatitis C (HCV) nucleic acid (DNA or RNA) and/or a positive antibody test were considered HCV co-infected. If no HCV testing was noted subjects were excluded.

We hypothesized that incorporating multiple biomarkers to describe the inflammatory profile of elderly HIV^neg^ subjects would better describe the inflammaged phenotype. K-means clustering was used to describe an inflammaging phenotype (IP) in HIV^neg^ subjects based on levels of iFABP, sCD14, sCD27, and hsCRP. The LPS and IL-6 markers were excluded from the analysis because these values were missing from 20% of the subjects. The WSS plot supporting a two-cluster solution is shown (Fig. 5A). Including a 3^rd^ cluster neither met the 40% reduction in WSS nor produced clinically relevant groupings (Fig. 5A). The mean biomarker levels were higher in IP+ subjects (Table S1). The clusters separated at approximately 60 years of age, although four young adults fell in cluster 2 (Fig. 5B).

A classification model based on the IP in HIV^neg^ subjects was applied to the HIV^pos^ subjects to determine whether HIV^pos^ subjects were IP+ before 60 years of age. The linear discriminate function was established in the HIV^neg^ subjects using the classification rule ± 60 years of age. The misclassification rate from cross validation was only 11% in HIV^neg^ subjects (data not shown). Although IP+ HIV^pos^ patients tended to be older (median: 60 years; 24–81) than IP- subjects (52 years; 29–69), the difference was not significant (*p = 0.098*), and both groups included subjects of all ages (Fig. 5C).

To evaluate whether HIV disease status was contributing to the IP+ or IP- classification, commonly measured clinical markers of HIV disease progression were compared between IP+ and IP- subjects. The CD4 count at draw, CD4 nadir, infection duration, and VACS index were not significantly different between IP+ and IP- HIV^pos^ subjects (Fig. 5 D–G). The viral load pre-cART; the frequency of pre-cART AIDS diagnosis (CD4 < 200 cells/mL); and the frequency of subjects with an immunologic non-responder (INR) status (CD4 < 350 cells/mL) were also not significantly different (data not shown). However, IP+ HIV^pos^ subjects had significantly higher LPS levels than IP- subjects, confirming that MT is increased in association with the IP (Fig. 5H). IP+ subjects also tended to have higher CMV-specific IgG titers (Fig. 5I; 1,274 EU/mL, 691 EU/mL; *p = 0.139*); a higher incidence of HIV/HCV co-infection (Fig, 5J; 26%, 7%; *p = 0.09*); and shorter treatment duration (data not shown; 51 vs 105 months; *p = 0.11*) suggesting increased exposure to viral antigens. The CMV IgG titer was significantly associated with both LPS (r =  0.260, *p =  0.026*) and sCD27 (r =  0.439, *p < 0.0001*) levels in the HIV^pos^ subjects (data not shown). These results suggest that underlying co-infections could be contributing to the inflammaging phenotype in the HIV^pos^ cohort.

**Figure 6:**
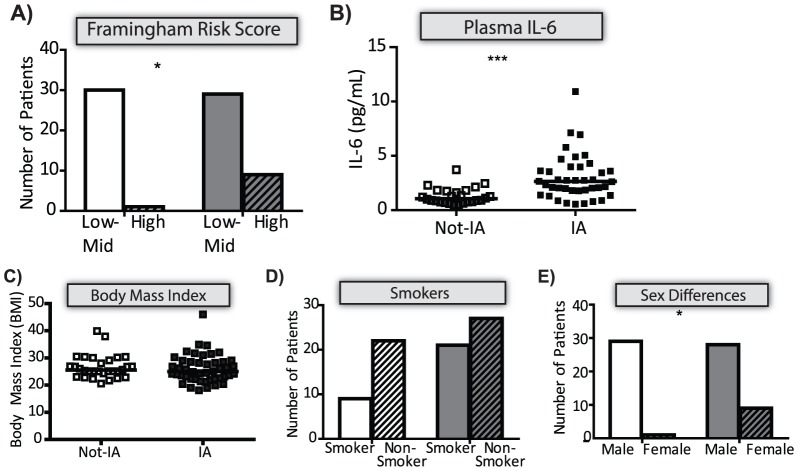
CVD risk factors in HIVpos subjects grouped by the IP. HIVpos subjects were classified as IP+ or IP- as described in Figure 5. In all panels, the subjects that were classified as IP+ are shown in closed squares or grey bars, while IP- subjects are shown in open squares or bars. (A) The risk of developing CVD was compared in IP+ and IP- subjects using the Framingham Risk Score. The number of patients falling in the low/mid risk and high risk groups are shown for IP+ and IP- subjects. Next, various CVD risk factors including: (B) Plasma IL-6 concentration; (C) Body Mass Index (BMI); (D) Smoker status, and (E) sex differences were compated between IP+ IP- subjects. To remain consistent with previous biomarker analysis: the IL-6 plasma concentration was natural log transformed and a T-test was used to determine statistical significance. The horizontal line indicates the geometric mean. In all other cases, statistical significance was determined using the Mann-Whitney U test or Fisher's exact test as appropriate, p-value < 0.05 was significant. (* - *p*  =  0.05–0.01; *** - *p* < 0.001).

Inflammation has a well-established link to developing cardiovascular disease (CVD). HIV^pos^ subjects were divided into groups of low to medium (1–20%) risk or high risk (>20%) of CVD using the Framingham index [23]. A significantly greater percentage of the IP+ HIV^pos^ subjects were at high risk of CVD (Fig. 6A) and had significantly increased levels of IL-6 (Fig. 6B). There were no significant differences in body mass index (Fig.6C) or smoker frequency (Fig. 6D) based on IP. However there were more women in the IP+ group (Fig. 6E). The use of statins, a class of frequently prescribed cholesterol lowering drugs that are anti-inflammatory, was not significantly different between IP+ and IP- subjects (data not shown; 29.2% vs 32.3%, p =  0.8059).

## Discussion

The gut is a prominent a source of persistent immune stimulation, particularly during HIV-1 infection. This study is one of the first to link IEBD and MT to markers associated with monocyte and T-cell activation in HIV^neg^ subjects. In addition, markers of IEBD, MT, and cellular immune activation were shown to increase with age similar to inflammatory biomarkers, leading to a more comprehensive definition of inflammaging. However, the etiology of gut damage in the aging HIV^neg^ population remains unclear. Primary IEBD could be triggered by age-associated vascular changes leading to ischemic damage and epithelial barrier leakiness [24]. Alternatively, IEBD could be the result of chronic subclinical mucosal inflammation. In baboons, the aging process causes decreased tight junction protein expression and increased levels of IL-1β, IFNγ, and IL-6, in the absence of overt inflammation by histology [24]. We have previously shown that low frequencies of resident lamina propria CD4+ T cells proliferate and produce IFNγ and IL-17 in response to commensal bacteria in an MHCII dependent manner [25], [26]. Cross reactivity between enteric pathogens (e.g. Salmonella) and commensal bacteria has been shown to cause accumulation of long-lived inflammatory T-cells [27]. These studies suggest that exposure to common enteric pathogens via MT may drive the expansion of long-lived inflammatory T-cells in the mucosa during normal aging.

Given the apparent similarities regarding IEBD, MT, and systemic immune activation that underlie inflammaging and HIV-1 pathogenesis, a key question in the HIV field is whether cART-treated, HIV-infected subjects develop an IP earlier in life. Our results indicate that although HIV^pos^ subjects can be IP+ as young adults, HIV infection does not fundamentally change the relationship between age and most of the inflammatory biomarker levels based on regression modeling. Although the aberrantly high levels of IEBD and MT markers that occur during HIV infection appear to mask more subtle age-associations, it is likely that MT still contributes to inflammaging in HIV^pos^ subjects.

The strong correlation between age and plasma sCD14 in HIV^neg^ subjects is consistent with previous work supporting a role for monocyte activation in inflammaging, although this remains controversial [6], [28]. Monocyte activation in HIV^neg^ elderly subjects has been associated with frailty [29] and increased risk of CVD, stroke, and mortality [6]. It is unclear whether aging changes the number of total circulating monocytes [30], but it is clear that CD14+CD16+ monocytes, which are pro-inflammatory and pro-atherosclerotic, increase in proportion from 3% to almost 20% in the elderly [31]. We have previously shown that peripheral CD16+ monocytes respond to commensal enteric bacteria by producing the pro-inflammatory cytokine IL-23 [32], [33]. In addition, CD16+ monocytes release IL-6, TNFα, and IL-8 after stimulation with bacterial derived TLR agonists (LPS, Pam3Cys, LTA, LPS and Flagellin) [30], [31]. Both IL-6 [34] and TNFα [35] cause skeletal muscle catabolism suggesting a mechanistic link between MT and frailty. CD16+ monocytes also potently induce T cell proliferation in response to bacterial toxins [31]. Indeed, we observed a strong association between sCD14 and sCD27 levels. Therefore, CD16+ monocytes could be contributing to elevated inflammation through innate and adaptive immune effector functions. Surprisingly, we found that sCD14 levels in the HIV^pos^ subjects did not reach the same level as the HIV^neg^ elderly. However, as expected, compared to the HIV^neg^ subjects, the levels of sCD14 were significantly elevated in younger HIV^pos^ subjects. Possible explanations for lower maximal levels of sCD14 in elderly HIV^pos^ subjects include: LPS tolerance from chronic exposure as a result of MT [36]; HIV-protein (Vpr, Tat) mediated alteration of the monocyte endotoxin response [37], [38]; and the HIV-associated monocyte subset switch to CD14-CD16+ monocytes resulting in fewer monocytes able to shed CD14 as disease progresses [39].

Given the heterogeneity of biomarker levels within an individual subject, we hypothesized that multiple biomarkers would better define an “inflammaged” phenotype than any single marker. The phenotype we defined included a traditional inflammatory biomarker (hsCRP) as well as biomarkers reflecting novel gut-related processes (iFABP, sCD14) and cellular immune activation (sCD14, sCD27). IP+ HIV^pos^ subjects identified using this approach represented the entire age range, suggesting that age is not a defining parameter of the IP during HIV infection. Similarly, common clinical markers of HIV disease progression, like peripheral CD4^+^ T cell count, were comparable between IP- and IP+ subjects. These findings suggest that in this cohort of HIV^pos^ subjects, neither age nor stage of HIV disease progression were driving classification in the IP+ group.

However, our results do suggest that persistent antigen exposure likely contributes to development of the IP during HIV-1 infection. HIV^pos^ subjects are exposed to persistent antigen from a variety of sources including: low level HIV replication despite cART, MT, and co-infections. Consistent with this hypothesis, IP+ subjects had significantly higher LPS levels than IP- subjects. This would suggest an association between MT and chronic bacterial antigen exposure that contributes to the IP. In addition, surrogate markers for viral pathogen exposure also tended to be elevated in the IP+ group. Persistent infection with CMV and EBV has been linked to the immune risk profile (IRP) in the HIV^neg^ elderly [1], [40]. In HIV-infection, asymptomatic CMV or EBV reactivation leads to CD8 T cell senescence 20–30 years earlier than in uninfected populations [41], and co-infection with HCV was associated with increased mortality [42]. These results suggest that exposure to viral and bacterial pathogens contribute to the development of the IP. The IP+ group also had a higher incidence of subjects at high risk of developing CVD. Treated HIV-infected patients have an elevated risk of atherosclerosis, myocardial infarction, and heart failure compared to uninfected peers for a multitude of reasons, including possible drug toxicity [43]. However, CVD risk has also been linked to pathogen exposure [44], elevated LPS levels [45], high CMV-specific T cell responses [46], and high CMV IgG titer [8], [47]. Additional studies are needed to determine whether qualitative differences in the type of pathogen exposure (e.g. predominately viral or bacterial, which pathogens or co-infections, etc.) impact the IP and risk of developing CVD.

The rapid development of innovative multiparameter technology has created an unprecedented opportunity to examine human biology in different clinical settings. The novel inflammaging signature we describe here was associated with increased CVD risk in HIV^pos^ subjects but was independent of most common HIV-1 disease parameters and some traditional CVD risk factors. We also report higher levels of LPS in subjects with the inflammaging signature, which has been linked to increased CVD risk in non-smoking HIV^pos^ subjects [45]. The type of aggregate analysis applied here is critical for measuring multi-system changes. In related studies, allostatic load, a cumulative measure of deregulation across multiple systems, was a better predictor of decline in high-functioning elderly participants in the MacArthur Studies of Successful Aging than any single biological parameter measured [48]. A second example, the immune risk profile (IRP), is characterized by CMV seropositivity, an inverted CD4:CD8 ratio, reduced T cell proliferative potential, and accumulated memory T-cells [40], [49], and is associated with increased risk of all-cause mortality [50]. Analyses leveraging multiparameter technology have also been used to distinguish HIV-infected controllers and non-controllers [51] and define a cytokine based immune activation signature [52] during HIV-infection. The advantage of the current work is that: (1) plasma biomarkers are easily measured, (2) continuous variables were used to generate the risk profile, and (3) it incorporated measures of change in multiple biological processes. Diagnostic platforms leveraging this technology to comprehensively define the IP could provide a cost effective and minimally invasive method of identifying patients at high risk of CVD and other adverse outcomes associated with inflammaging.

A strength of this study is that we were able to describe inflammaging over the entire adult lifespan, incorporating six biomarkers that reflect different mechanistic pathways of inflammation. Due to the retrospective nature of the study, there were imbalances between the groups including age, sex, and race, which may have confounded our interpretation. The age distribution differences between the HIV^neg^ and HIV^pos^ cohorts do not impact the conclusions about inflammaging based on analyses within each cohort. However, they may limit our ability to draw conclusions based on direct comparisons between the HIV^neg^ and HIV^pos^ cohorts. Another limitation of this study is that the small sample size of subjects with extreme longevity was insufficient to address unique biomarker expression patterns in this age group. Sex differences in age-associated sCD14 and monocyte function have been described previously [6], [28], and the preponderance of men in the study likely masked any of those differences in our cohorts. There are conflicting reports regarding whether race, or race-associated confounders (e.g. BMI), impact the concentration of plasma hsCRP and IL-6 [4]. However, elevated levels of hsCRP and IL-6 were associated with poor prognosis regardless [4], suggesting that racial differences were less likely to impact the conclusions in our study. The study is also limited by its cross sectional nature and the lack of detailed medical information for the HIV^neg^ participants. Carefully controlled prospective studies using age and risk matched cohorts are required to confirm and extend the findings from this study.

## Methods

### Ethics Statements

This study was approved by the Colorado Multiple Institutional Review Board (COMIRB), and all subjects provided written informed consent.

### Study Design

A cross sectional retrospective study was performed utilizing 171 plasma samples from adult donors 20–100 years of age. Patients were stratified as young adults (18–39 years), middle-aged (40–59 years), or elderly (60+ years). Self-identifying HIV^neg^ subjects were recruited at the University or through the University of Colorado Hospital (UCH) Internal Medicine Clinic. Due to the retrospective nature of the study, the HIV^neg^ subjects were recruited under a consent process that did not include access to medical records. HIV-infected cART-treated patients (HIV^pos^) with undetectable viral loads (<48 copies/mL) for at least 6 months were recruited from the UCH Infectious Diseases Group Practice Clinic. Patients that were pregnant or breast feeding, or had active infections were excluded.

### Medical History

HIV^pos^ subjects' medical records were reviewed for: gender; ethnicity; HIV-associated clinical parameters (CD4 count, viral load, ART regimen); routine laboratory values (hemoglobin, AST, ALT, platelet count and creatinine levels); height, weight or body mass index (BMI); smoking status; statin use; and lipid levels (cholesterol, HDL). The time between the draw date and HIV diagnosis or first exposure to cART was estimated from notes. The BMI, Veterans AIDS Cohort Study (VACS) score, and Framingham Cardiovascular Disease Risk Score were calculated from clinical values.

### Plasma Biomarker Assays

Venous blood was drawn into BD Vacutainer tubes containing EDTA (LPS, sCD27, IL-6) or sodium heparin (iFABP, sCD14, hsCRP, CMV IgG). The plasma was separated by centrifugation at 700xG for ten minutes and stored at −80°C. ELISAs were conducted per the manufacturers' protocols: LPS (dilution 1∶10; limulus assay, Lonza); sCD27 (1∶20; Cell Sciences); IL-6 (1∶1; R&D; high sensitivity kit); iFABP (1∶500; R&D Duoset); sCD14 (1∶500; R&D Quantikine kit); hsCRP (1∶5,000; R&D Duoset); and CMV specific IgG (1∶1,000; Diamedix). An internal control sample from an HIV^neg^ subject was included in each assay. The conditions for the LPS assay were empirically optimized, and a 1∶10 dilution of plasma was chosen to maximize assay sensitivity and minimize plasma inhibition. The coefficients of variability determined from the control sample from at least four runs were: LPS-32.41%; sCD27-4.56%; IL-6-0.21%; iFABP- 3.37%; sCD14- 4.59%; and hsCRP- 0.21%.

### Statistical Analysis

#### Grouped analyses of Plasma Biomarkers

Grouped data were analyzed using ANOVA and *t*-tests on natural log transformed biomarker values. The Benjamini-Hochberg method was used to control the false discovery rate (FDR) at the 0.05 level with multiple comparisons [53]. The results are presented on the untransformed scale to show the plasma concentration of each marker. No outliers were identified in the natural log transformed data used for statistical analysis despite the apparent skew on the untransformed scale.

#### Regression models and whole cohort analyses

The plasma concentration of the plasma biomarkers was natural log transformed for all regression analyses and for the subsequent k-means clustering and classification model. Associations between age and plasma levels of biomarkers associated with IEBD (iFABP), direct (LPS) and indirect (sCD14) markers of MT, T cell activation (sCD27), and systemic inflammation (hsCRP, IL-6) were evaluated using Pearson correlations. sCD14 also reflects monocyte activation [28]. Cluster version 3.0 (http://rana.lbl.gov/EisenSoftware.htm) was used to visualize the untransformed plasma levels of the biomarkers as median centered heat maps [51] in Java TreeView (http://jtreeview.sourceforge.net/docs/overview.html) version 1.16r2 [54]. Regression model 1 was fit to the HIVneg cohort using the covariates age and sex to determine whether sex was influencing the relationship between age and each biomarker in this study. Regression model 2 was fit to both the HIVneg and HIVpos cohorts using the covariates age, HIV-infection status, and allowing for an HIV/age interaction term to determine whether HIV-status altered the relationship between age and each biomarker. For the biomarkers where the HIV/age interaction term was not significant (lines had similar slopes), we used additional regression analyses (model 3) to determine the average number of years it would take for an HIV^neg^ subject to develop biomarker levels equivalent to an HIV^pos^ subject. In other words, the average difference between the biological age of an HIV^pos^ subject, measured using biomarker levels, and their chronological age was approximated. The y-intercept difference (the average difference in biomarker expression levels between HIV^pos^ and HIV^neg^ subjects of the same age) was divided by the slope of the regression line (the average increase in biomarker levels for each year of age).

To describe inflammaging using aggregate biomarker expression k-means clustering was used to identify patterns in biomarker expression regardless of actual age. K-means clustering was used to determine whether there were HIV^neg^ subjects that had similar patterns of plasma biomarker levels regardless of their age. This method is an iterative unsupervised approach to exploring patterns in data. Data points are assigned to the nearest cluster center and the center is recalculated until it does not move. This process is repeated using a variable number (k) of clusters. To determine the most appropriate number of clusters in the dataset the within clusters sum of squares (WSS) is used to assess the homogeneity of each cluster in the final solution for each k value. The more similar the points making up the cluster are to each other the smaller the WSS value will be. In practice, a significant reduction of 40% or more in the WSS value justifies the addition of another cluster to the solution. K-means clustering was used to describe patterns of expression in 4 of the 6 biomarkers: iFABP, sCD14, sCD27, and hsCRP. LPS and IL6 were excluded from the model because approximately 20% of the subjects were missing values for these parameters. In exploratory analyses using only subjects with complete biomarker panels, including LPS and IL6 in the model did not significantly alter the results of the k-means clustering (data not shown). However, the substantial decrease in subject number was considered detrimental to the precision of the classification model (discussed below) and downstream analyses lost power. Therefore, we chose to use only iFABP, sCD14, sCD27, and hsCRP to define inflammaging.

Partially based on the results of the k-means clustering analysis, a classification model was developed using the HIV^neg^ cohort where subjects were classified as either “IP+” or “IP-” based on a cutoff of 60 years of age. A linear discriminant function was fit using the plasma biomarkers sCD14, sCD27, CRP and iFABP. Leave-one-out cross validation was used to evaluate the model's performance. The resulting model was then applied to the HIV^pos^ subjects to determine whether HIV^pos^ subjects were IP+ before 60 years of age. Statistical analysis was conducted using GraphPad Prism version 5.04 (San Diego, CA) and R version 2.15.3.

## Supporting Information

Figure S1 **Age Distribution in the HIVneg and HIVpos cohorts.** The ages of the subjects enrolled in the HIVneg and HIVpos cohorts are shown. A) The age for each individual is shown. The horizontal line represents the median age of the cohort. HIVneg subjects are shown in open circles and HIVpos subjects are shown in closed circles. B) The age distribution for each cohort is shown by decade. The Y-axis value is the center of the decade (e.g. subjects in the category 50 are between 45–54. years of age). The percent of each cohort falling in a given decade is shown on the X-axis. HIVneg subjects are shown in white bars and HIVpos subjects are shown in black bars.
(EPS)

Figure S2 **Comparing the plasma concentration of IEBD, MT, and inflammatory markers in HIVneg subjects stratified by age.** The biomarker concentrations were natural log transformed and compared between groups using an ANOVA, a p-value < 0.05 was considered significant. The Benjamini-Hochberg method was used to control false discovery rate (FDR) at the 0.05 level. The horizontal line indicates the geometric mean of each group. The fold-change is indicated when differences between the groups were significant. N.S. – not significant. * −0.05–0.01; **−0.01–0.001, ***−< 0.001. Each group is represented by the same symbol throughout the figure: young adult HIVneg subjects - circle; middle age HIVneg - square, elderly HIVneg – triangle. iFABP- top left, LPS- top right, sCD14- middle left, sCD27- middle right, hsCRP- bottom right, and IL-6- bottom left.
(EPS)

Figure S3 **Comparing the plasma biomarker concentration in age-stratified HIVneg and HIVpos subjects.** The plasma concentration of each biomarker is presented for subjects stratified into two age groups: young adult- 18–39 years of age and middle aged- 40–59 years of age. The biomarker concentrations were natural log transformed and compared between age matched groups using a t-test, a p-value < 0.05 was considered significant. The horizontal line indicates the geometric mean of each group. The fold-change is indicated when differences between the groups were significant. * −0.05–0.01; ** −0.004–0.006, *** - *p*  =  0.0008; **** - *p* <0.0001. Each group is represented by the same symbol throughout the figure: young adult HIVneg subjects - open circle; middle age HIVneg – open square, HIVpos young adults – closed circle, and middle age HIVpos- closed square iFABP- top left, LPS- top right, sCD14- middle left, sCD27- middle right, hsCRP- bottom right, and IL-6- bottom left.
(EPS)

Table S1 **Statistical Analyses Summary.**
(DOCX)

Table S2 **Regression Summary Tables.**
(XLSX)
