# Supplementary material for: Contribution of Intestinal Barrier Damage, Microbial Translocation and HIV-1 Infection Status to an Inflammaging Signature
Source: PLoS One. 2014 May 12;9(5):e97171. doi: 10.1371/journal.pone.0097171 (PMC4018269; doi:10.1371/journal.pone.0097171)
Supplement: Table S1 — Statistical Analyses Summary. (DOCX) [file pone.0097171.s004.docx]

| **Supplemental Table 1: Statistical Analyses Summary** | | | | | | |  |  |
| --- | --- | --- | --- | --- | --- | --- | --- | --- |
| **REGRESSION MODELS:** | | | | | | | |  |
|  | **Model 1** | | | | **Model 2** | | **Model 3** |  |
|  | ***Age^1^*** | | ***Sex (M)^1^*** | | ***HIV/age Interaction^2^*** | | ***Age Difference (years)^3^*** |  |
| ***Biomarker*** | ***Coefficient*** | ***p-value*** | ***Coefficient*** | ***p-value*** | ***Coefficient*** | ***p-value*** |  |  |
| LPS | 0.0064 | **0.0415** | 0.3495 | **0.0129** | 0.00097 | 0.867 | 6.1 |  |
| sCD14 | 0.0075 | **<0.0001** | -0.0518 | 0.2088 | -0.00608 | **0.003** | n.d^4^ |  |
| sCD27 | 0.0126 | **<0.0001** | 0.127 | 0.0945 | -0.00186 | 0.5996 | 16.8 |  |
| iFABP | 0.008 | **0.0051** | -0.1084 | 0.3533 | -0.00704 | 0.2444 | 141.3 |  |
| CRP | 0.0165 | **0.0133** | 0.0677 | 0.8054 | 0.00156 | 0.8832 | 38 |  |
| IL-6 | 0.0201 | **<0.0001** | -0.0264 | 0.8762 | 0.00602 | 0.3771 | 15.8 |  |
|  |  |  |  |  |  |  |  |  |
| **k-MEANS CLUSTERING SOLUTIONS- GROUP MEANS** | | | | | | |  |  |
| ***Group*** | ***age*** | ***iFABP (pg/mL)*** | ***sCD14 (pg/mL)*** | ***sCD27 (pg/mL)*** | ***hsCRP (ug/mL)*** |  |  |  |
| 1 | 35.7 | 1490 | 1.90E+06 | 192 | 1.63 |  |  |  |
| 2 | 64.1 | 2610 | 2.60E+06 | 379 | 2.06 |  |  |  |
| ^1^ regression model with age and sex as covariates | | | | | | | | |
| ^2^ regression model with age, HIV status, and an HIV-age interaction term | | | | | | | | |
| ^3^ For biomarkers where there was not a significant interaction between HIV-status and age, separate regression models were fit with only HIV-status and age to determine how much "older" the HIV-infected patients appear. | | | | | | | | |
| ^4^ not determined because the interaction between age and HIV-status was significant for sCD14 | | | | | | | | |
